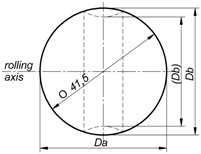# The Effect of Process Parameters in Helical Rolling of Balls on the Quality of Products and the Forming Process

**DOI:** 10.3390/ma11112125

**Published:** 2018-10-29

**Authors:** Janusz Tomczak, Zbigniew Pater, Tomasz Bulzak

**Affiliations:** Faculty of Mechanical Engineering, Lublin University of Technology, 36 Nadbystrzycka Str., 20-618 Lublin, Poland; z.pater@pollub.pl (Z.P.); t.bulzak@pollub.pl (T.B.)

**Keywords:** helical rolling, balls, grinding media, fem

## Abstract

This paper presents selected numerical and experimental results of a skew rolling process for producing balls using helical tools. The study investigates the effect of the billet’s initial temperature on the quality of produced balls and the rolling process itself. In addition, the effect of billet diameter on the quality of produced balls is investigated. Experimental tests were performed using a helical rolling mill available at the Lublin University of Technology. The experiments consisted of rolling 40 mm diameter balls with the use of two helical tools. To determine optimal rolling parameters ensuring the highest quality of produced balls, numerical modelling was performed using the finite element method in the Forge software. The numerical analysis involved the determination of metal flow kinematics, temperature and damage criterion distributions, as well as the measurement of variations in the force parameters. The results demonstrate that the highest quality balls are produced from billet preheated to approximately 1000 °C.

## 1. Introduction

One of the most efficient methods of manufacturing balls for ball mill grinding media is skew rolling with helical tools. The concept of the rolling process for producing balls with the use of helical tools was developed at the turn of the 19th and 20th centuries [1,2,3,4]. However, the intensive development of this method did not take place until in the 1950s, when the OAO EZTM (Moscow) industrial plant in the former Soviet Union launched the industrial production of balls by helical rolling [5,6,7]. The solutions developed at that time are still used today. At present, the helical rolling technique for producing balls is widely used primarily in Russia and China.

Balls are produced in skew rolling mills provided with two rolls with helical grooves usually made at the length of 3.5 coils. The axes of the rolls are inclined askew relative to the axis of the billet material (rod), usually at an angle ranging from 3° to 7°. During rolling, the rolls are rotated in the same direction and make the working tool cut into the billet; the forming flanges rotate the material in an opposite direction to the rolls and shift the billet axially inside the roll gap. To ensure good rolling results, the diameter of the billet should be approximately 3–4% smaller than the diameter of the balls. However, the diameter of the rolls should be 5–6 times bigger than the diameter of the balls [8,9,10,11,12,13,14].

Although the helical rolling method for producing balls has been used for quite a long time, the literature of the subject lacks detailed studies on tool design and the effect of applied operational parameters on the rolling process and the quality of produced balls. On the other hand, industrial solutions in the field of helical rolling, often developed by trial and error, are treated as commercial secrets. Therefore, it is justified to conduct research that will enhance the current state of knowledge in this area.

One of the parameters affecting rolling processes for producing balls is the temperature of a billet. In helical rolling, balls are formed in hot working conditions from a material with increased contents of carbon and manganese. Immediately after rolling, the balls are subjected to quenching. As a result, the temperature of the balls after the rolling process should be high enough to ensure that they can be quenched without an additional heating operation. The other essential parameter influencing the quality of balls produced by helical rolling is the diameter of a billet material. The rolling process for balls involves the use of semi-finished products in the form of hot-rolled rods with relatively high dimensional tolerances (the tolerance range for these rods often exceeds 1.5 mm). Therefore, properly calibrated tools should provide a possibility of forming balls with the required accuracy from semi-finished products with extreme dimensional tolerances.

## 2. Object of the Study

**Figure 1:**
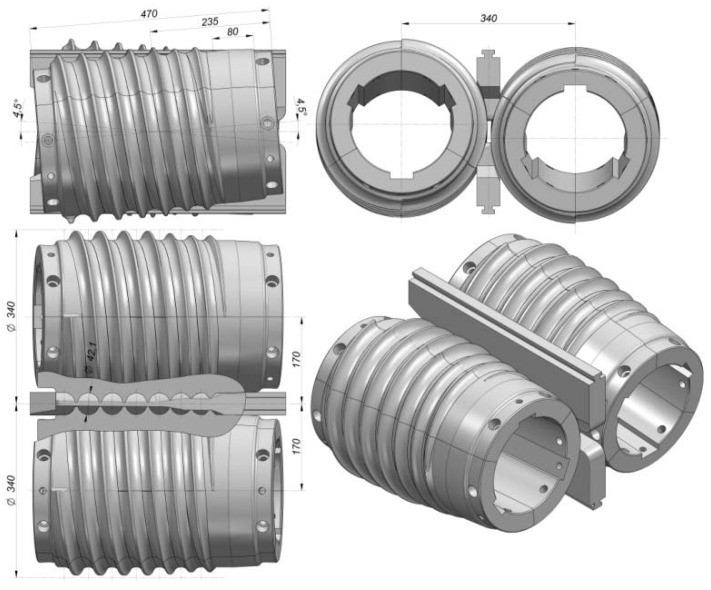
Shape and position of the helical tools used for rolling 41.5 mm-diameter balls.

The effect of operational parameters was investigated in experimental testing of a helical rolling process for producing 41.5-mm-diameter balls. A set of helical tools was designed specifically for the purpose of this study. The tool set consists of two rolls with segmented design, two guides and a billet positioning sleeve. On their working surface, the rolls have helical grooves, the profile of which corresponds to that of the balls. The grooves form a circular roll pass. During the process, the axes of the rolls are twisted in opposite directions relative to the axis of rolling at the same angle *γ*, which is set equal to 4.5°. The shape and position of the tool during rolling are shown in Figure 1. During the rolling process, the rolls are rotated in the same direction at the same velocity, which is set equal to *n* = 30 rev/min. The billet material was C55 rods with diameters of 41 mm, 40 mm and 39 mm, and a length of *l* = 350 mm. The experiments were performed for four values of initial temperature of the billet: 950 °C, 1000 °C, 1050 °C, and 1150 °C, respectively.

## 3. Test Stand and Experimental Tests 

The rolling tests were performed in a skew rolling mill designed at the Lublin University of Technology [15] and installed at the Centre for Innovative Technologies of the Lublin University of Technology. This skew rolling mill has a modular design, consisting of a support frame, a drive system, a mill stand and a drive transmission system. The machine frame is an openwork structure made from welded thin-walled sections. The drive system consists of an electric motor, a belt transmission and a gear transmission, and it is attached to the frame. The mill stand is mounted on the opposite side of the frame, and it contains the working rolls and tools, as well as the guides for positioning the billet in the machine work space and the equipment for setting roll position. The position of the working rolls can be set within a fairly wide range, which increases the technological capabilities of the machine. In addition, the drive system is equipped with a two-speed motor, which makes it possible to increase the throughput of the process when producing small parts.

**Figure 2:**
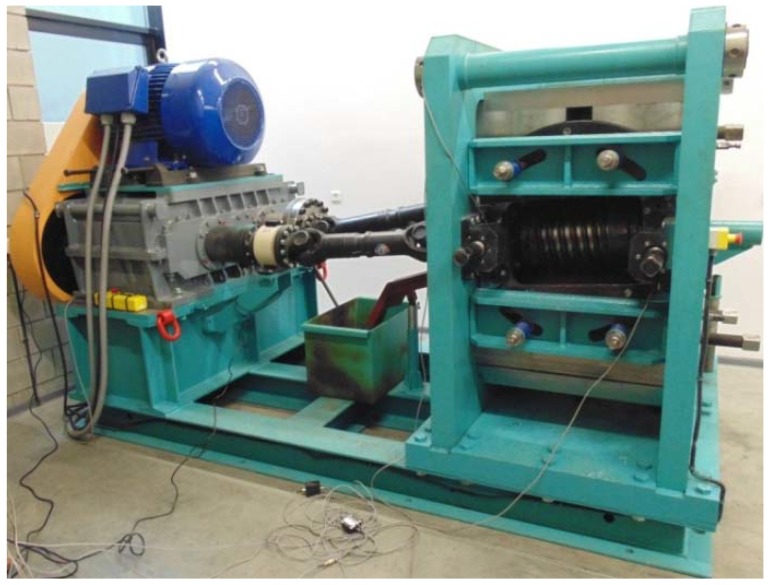
Helical rolling mill used in the experimental tests.

The transfer of torque from the drive system to the main shafts of the roll stand is carried out via articulated shafts. In addition, the angular position of one of the working shafts can be continuously adjusted, which makes it much easier to set the tools after replacement. The rolling mill is also equipped with torque and pressure measuring systems that communicate with the machine control system and protect the drive system and tools from overload and damage. The technical specifications of the rolling mill are given in Table 1, and the rolling mill is shown in Figure 2.

**Figure 3:**
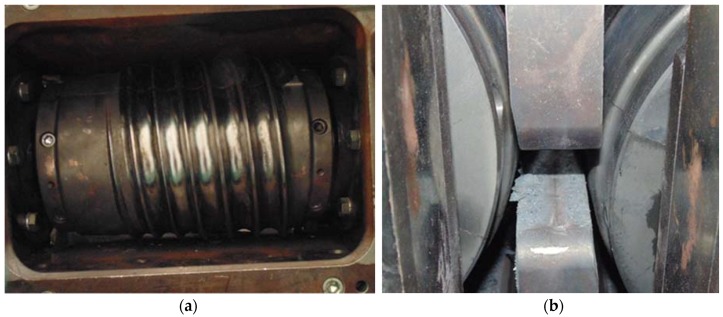
Tools for forming balls mounted in the skew rolling mill: (**a**) helical roll; (**b**) upper and lower guides.

For the purpose of the experiments, two sets of helical tools were mounted on the rolls (in accordance with Figure 1). Each tool set consisted of three individual segments mounted every 120° on the circumference of the shafts. One of these helical tool segments is shown in Figure 3a. In addition, two guides (upper and lower) were installed between the rolls to ensure that the billet is in the tool working space during rolling (Figure 3b).

**Figure 4:**
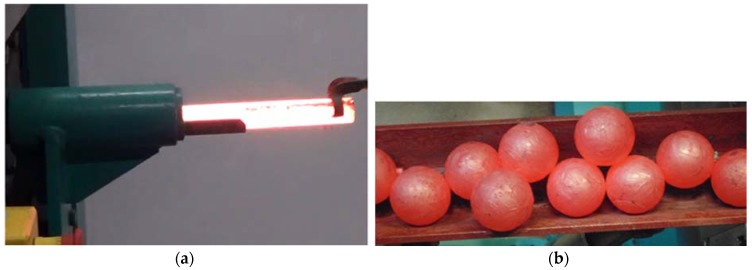
Rolling of balls in a helical rolling mill: (**a**) billet is fed into the machine work space via sleeve; (**b**) balls are removed from the machine work space via receiving chute.

Prior to rolling, the billet material (rod) was pre-heated in an electric chamber furnace to the assumed forming temperatures. After that, it was placed in the positioning sleeve and fed into the working space of the machine (Figure 4a). During rolling, the billet was rotated by the wedge flanges of the rotating rolls and automatically drawn into the helical roll passes formed by the opposite flanges of the tools. The forgings of balls were formed by the tools in a continuous manner until the entire billet had been used. The produced balls were automatically removed from the machine working space via the receiving chute (Figure 4b).

## 4. Experimental Results

**Figure 5:**
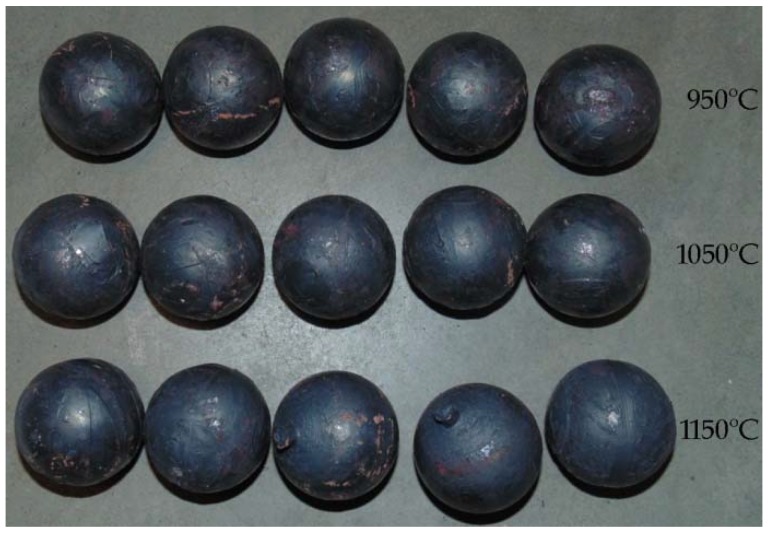
Shape of balls formed from 40 mm-diameter billet of varying initial temperatures.

**Figure 6:**
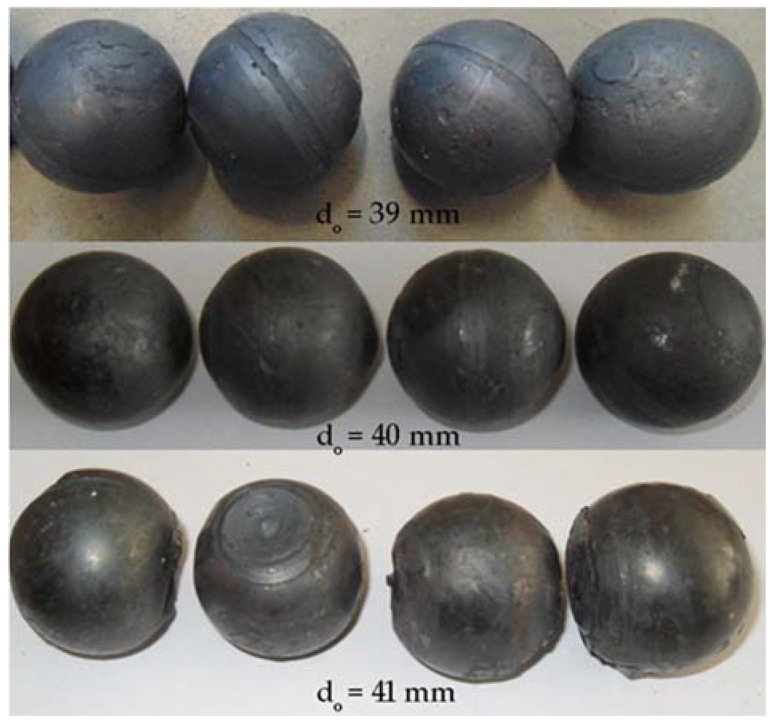
Shape of balls formed from billet material of varying diameters preheated to 1000 °C.

In the experiments, six batches of balls were produced, and their examples are shown in Figure 5 and Figure 6. According to the adopted experiment design, the tests were carried out for different initial temperatures and different diameters of the billet. As can be seen in Figure 5, the balls formed from the 40 mm diameter rods in the entire tested temperature range are characterised by a relatively accurate and spherical shape of the surface. However, in the case of balls formed from the billet preheated to 1150 °C, one can notice that the connecting necks are not completely separated from the surface of the balls. The produced balls either still have a connector on one side (Figure 5) or they are still connected to each other by the connectors. This phenomenon is not observed for balls produced from the billet preheated to lower temperatures. The incomplete separation of the connectors results from the employed method for separating balls. In traditional tool solutions, the balls and connectors are cut off by means of special cutters mounted on the end coils of the flanges. However, given the short service life of the cutters, a different separation method was used. According to the proposed concept, the cutters were not used; instead, the number of coils on the calibrating flanges increased and different outside diameters of the two rolls were used. In effect, the balls could be separated as a result of the differences in the tangential speeds (by twisting), while the connector was broken during the automatic revolution of the ball caused by the difference in diameters of the rolls. As demonstrated by the results (Figure 5), this solution ensures effective rolling of balls from the billets with lower preheating temperatures. However, the increase in the preheating temperature leads to an increase in the plasticity of the material, making it difficult to separate the balls and remove the connectors. The tests also involved the investigation of the effect of billet diameter on the rolling process and the quality of produced balls. The rolling tests were carried out for three diameters of the billet: 39 mm, 40 mm and 41 mm, assuming the same preheating temperature of 1000 °C. The obtained results (Figure 6) demonstrate that the balls formed from the 39 mm-diameter rods have a slight circumferential underfill of the spherical profile. The underfill has the shape of a ring-shaped groove located in the axis perpendicular to the axis of rolling. This indicates that the amount of material constrained in the helical roll pass was insufficient. The highest quality is exhibited by the balls formed from the 40 mm-diameter rods. These balls have a smooth and spherical surface, and are free from any defects. The use of a larger diameter billet (41 mm) causes overflow, resulting in both significant ovalisation of the cross section of the balls and flattening of the spherical profile along the rolling axis. It was also observed that the overfill may lead to the occurrence of cracks in the central region of forged balls (along the rolling axis), which often disqualifies such balls.

The balls formed in each test series were examined for their geometric accuracy. The obtained results are summarised in Table 2. The measurements were made in two planes: along the rolling axis (*D_a_*) and along the axis perpendicular to the rolling axis (*D_b_*). The results indicate that the dimensional accuracy of the produced balls is very high. In the case of balls formed from the 40 mm-diameter rods, the tolerance range is +/− 0.2 mm. However, in the case of the balls formed from the billet preheated to higher temperatures, the connectors are not completely separated. The use of a smaller-diameter billet (39 mm) leads to producing relatively accurate balls (despite the presence of an approximately 0.3 mm deep ring-shaped groove on the tool circumference). The geometric accuracy of balls from the 41 mm-diameter billet is much lower. One can see that the balls are visibly flattened in the rolling axis even as much as 4.4 mm, which exceeds the acceptable deviation for balls used as grinding media [16].

**Figure 7:**
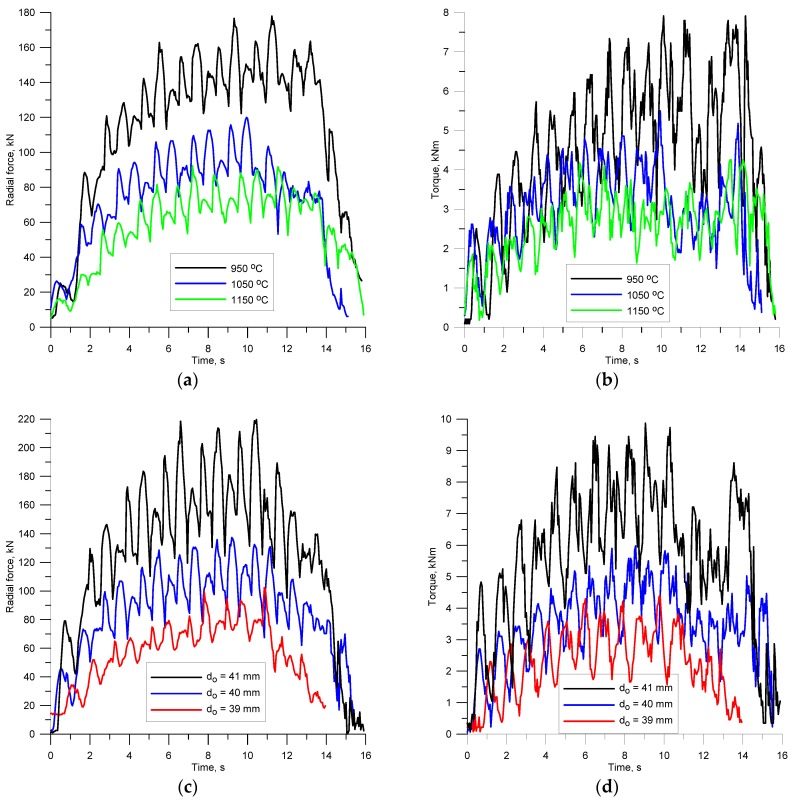
Variations in the force parameters in helical rolling of balls: (**a**) temperature versus radial force when billet diameter is 40 mm; (**b**) temperature versus torque when billet diameter is 40 mm; (**c**) billet diameter versus radial force when preheating temperature is 1000 °C; (**d**) billet diameter versus torque when preheating temperature is 1000 °C.

One of the key aspects of every process design is the ability to accurately determine force parameters of the process. The knowledge of behaviour patterns of the force and torques allows the correct design of tool shape and the selection of the rolling mill size. Based on variations in the force parameters, one can also predict phenomena that disturb stability of the process. For this reason, the radial forces and torques were measured during rolling (Figure 7). A characteristic of their behaviour is periodicity, which is directly associated with the nature of the investigated rolling process. Every half revolution of the tools, the first helical coils cut into the billet and separate a material volume that is equal to the volume of one ball. This leads to a periodic increase in the forces and torques. Then, in a further part of the helical roll pass, the workpiece is gradually shaped into a ball, as shown by the decreasing values of the forces and torques.

The amplitude of variations in the extreme values of the forces and torques strongly depends on both the preheating temperature of the billet and its diameter. It is obvious that as the temperature drops, the forces and torques increase. The results show that decreasing the billet temperature by about 100 °C leads to an approximately 50% increase in the forces and torques (Figure 7a,b). One can also observe an increase in the amplitude of the variations in the forces and torques. The variations in the radial force amplitude are relatively small, while the amplitude of the torque variations visibly increases with decreasing the billet temperature. The change of the billet diameter also has a significant effect on the behaviour pattern of the force parameters. However, in this case, the increase in the force parameters caused by the change of the billet is more significant (Figure 7c,d). On increasing the diameter of the billet merely by 1 mm, there is a nearly two-time increase in the forces and torques. Interestingly, the increase in the diameter of the billet leads to a visible increase in the amplitude of variations in both the forces and torques. Therefore, by monitoring the force parameters during rolling, one can predict the occurrence of various types of undesired phenomena such as overfill or underheating.

**Figure 8:**
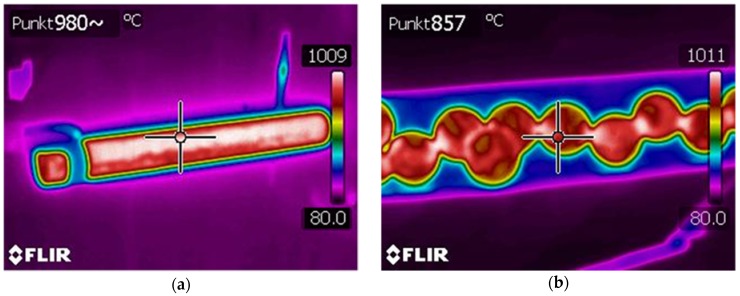
Thermograms from thermal imaging camera of: (**a**) billet; (**b**) formed balls.

To increase their hardness and resistance to abrasion, balls used as grinding media in ball mills are subjected to thermal treatment (quenching). An effective process for manufacturing balls should allow for the quenching of produced balls immediately after rolling, without the necessity of additional heating or slow cooling to achieve the required thermal treatment temperature. To determine the variations in the temperature of the material during the rolling process, thermal parameters of the preheated rods and the formed balls were analysed. The temperatures of the billet and the produced balls measured with a thermal imaging camera are shown in Figure 8. As expected, the temperature of the workpiece decreases during the rolling process, which is caused by the transfer of heat to the tools and the environment. It can be observed that the surface temperature of the balls produced from the rods preheated to 1000 °C is about 860 °C, which allows for the quenching of balls immediately after the rolling process. Locally, in the regions of the largest cross-sectional reduction, the temperature increases above its initial value, which is caused by the change of plastic deformation work into heat. To obtain homogeneous strength properties, balls should therefore be left for a few seconds in air prior to quenching, as this will help equalise the temperature in the entire volume of the material.

## 5. Numerical Modelling of the Helical Rolling Process for Producing Balls

**Figure 9:**
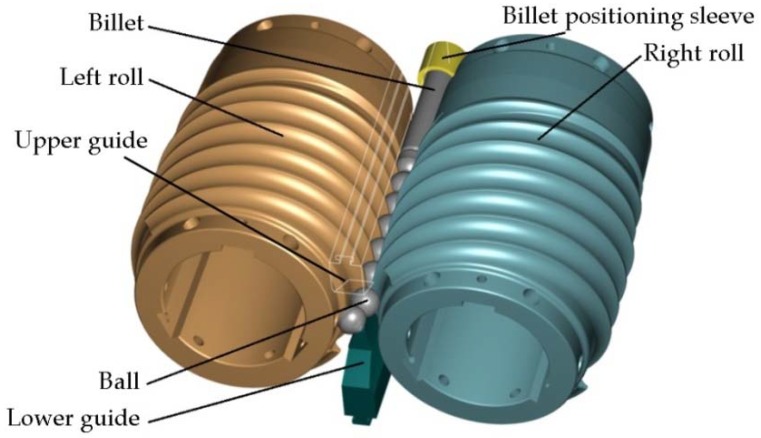
Geometric model of a helical rolling process for producing balls.

For optimal process parameters, yielding the highest quality rolled parts, numerical simulations were performed by the finite element method using the Forge NxT software (ver. 1.1, Transvalor, Mougins, France). The simulations of the rolling process were performed in a three-dimensional state of strain, taking account of thermal phenomena. The simulations investigated a rolling process for forming of balls from 40 mm-diameter rods preheated to the temperature of 1000 °C. For the purpose of the analysis, a geometric model of the rolling process was created, as shown in Figure 9. The model consisted of two rolls, two guides, billet positioning sleeve and billet. To minimise the calculation time, the tools were modelled as rigid objects. The tool design and kinematics of their movement reflected the parameters applied in the experimental tests. The billet was modelled as a rigid-plastic object using first-order, four-node tetragonal finite elements [17,18]. The temperature of the tools was maintained constant during the rolling process at 50 °C. The contact surface of the formed material and tools was described by the constant friction model represented by the friction factor equal 0.8. According to the dependence:

(1) $$\tau =m\cdot \frac{\sigma_{i}}{\sqrt{3}}\cdot \frac{\Delta v}{\left|\Delta v\right|},$$

where *τ* is the tangent stress on contact surface, MPa; *m* is the friction factor; *σ_i_* is the effective stress, MPa; and Δ*v* is the slip velocity on contact surface, mm/s.

As for other parameters used in the numerical modelling, the heat transfer coefficient between the material and the tool was set equal to 5 kW/m^2^K, whereas that between the material and the environment was 0.35 kW/m^2^K. The billet was assigned the properties of C55 steel, the model of which was obtained from the material model library of the applied software [19], it was defined by the following dependence:

(2) $$\sigma_{p}=1706.9923\cdot e^{-0.0028T}\cdot \varepsilon^{-0.19371}\cdot e^{-0.07421/\varepsilon }\cdot {\dot{\varepsilon }}^{0.1467},$$

where *T* is the temperature (ranging from 642.9 °C to 1250 °C), *ε* is the strain, $\dot{\varepsilon }$ is the strain.

**Figure 10:**
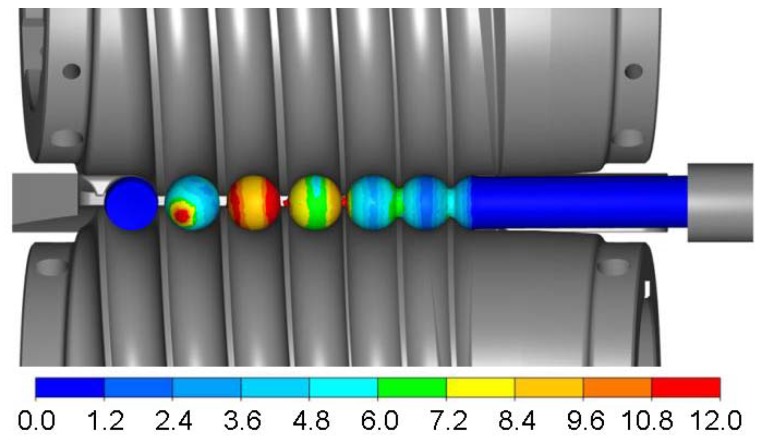
Finite Element Method-simulated changes of workpiece shape in helical rolling and the distribution of effective strains.

The numerical modelling involved the determination of material flow kinematics, the distributions of effective strains, temperatures, damage criterion as well as variations in the force parameters. The changes in the shape of the workpiece during the rolling process and the distribution of effective strains are shown in Figure 10. In the initial stage of the rolling process, the helical flanges on the tools rotated in the same direction cut into the billet periodically (every half revolution), making it rotate and constraining a volume of the material equal to the volume of the ball in the roll pass. At this time, a ring-shaped groove with flanks is formed on the circumference of the billet. In successive stages of the process, these flanks are shaped into ball bowls under the action of the helical flanges with concave surfaces. It takes two revolutions of the tools to produce a fully formed ball that is separated from the billet. Another 1.5 revolutions of the tools serves to calibrate the shape of the balls. Similar to the experimental tests, the separation of produced balls results both from the concentration of plastic strains in the connecting necks which are made narrow by the helical tools flanges and from the difference in the tangential speed in successive regions of the workpiece. It can be seen that for the applied process parameters, the numerically simulated shape of the balls is correct, free from surface defects and irregularities. The material adheres at every point to the lateral surfaces of the helical tools, which is a prerequisite for obtaining high geometrical accuracy. Therefore, it can be concluded that the boundary conditions adopted for calculations reflect real conditions.

Smaller values of effective strain in the first rolled ball result from reducing the material volume during the rolling process. This phenomenon is caused by extruding the material up to minimal waste between working collars of the tools during the shaping process of spherical surfaces of the product. As a result, a decrease in the material volume leads to lower effective strain (mostly in surface layers of the ball). Subsequent balls are rolled with the assumed material volume (uncontrollable displacement of the material is impossible due to the previous helical impression being filled with the material), which influences the growth of the strain values (observed values will be adequate for the next rolled products). A similar case will occur during the rolling process of the last ball, where part of the material will be extruded up to the minimal waste, therefore lowering the material volume of the last product, causing the strain value to decrease.

**Figure 11:**
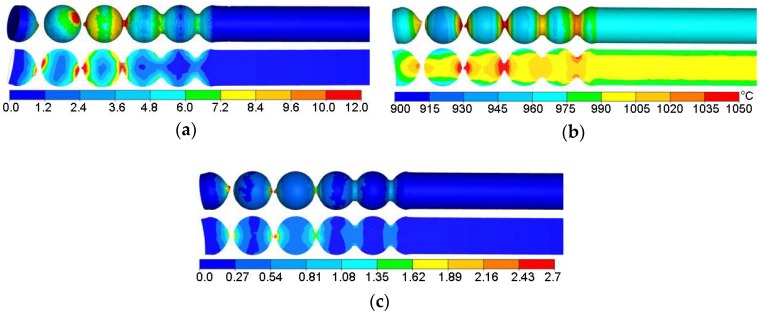
FEM-simulated distribution of: (**a**) effective strains; (**b**) temperature; (**c**) Cockcroft-Latham ductile damage criterion.

Figure 11 shows the distributions of effective strains, temperatures and the Cockcroft-Latham ductile damage criterion measured on the surface and in the axial sections of the workpiece. The highest plastic strains are located in the region of the connecting necks (Figure 11a). This phenomenon is characteristic of skew rolling processes for balls and is associated with the kinematics of material flow in the helical roll pass. The spherical shape of the balls is a result of gradual narrowing of the necks due to the action of the working flanges on the tools until complete separation of the material. In contrast, the strains in the workpiece centre are several times lower. This phenomenon is characteristic of rotary metal forming processes. The material undergoes higher deformation at the surface than in the centre. This is associated with the kinematics of the investigated rolling process which is characterised by large differences in the tangential speed of the workpiece (due to the changes in the tool pass radius). Consequently, there occur significant slips between the workpiece and the tools, and considerable strains are generated in a circumferential direction (by friction forces).

During the numerical simulations, thermal parameters of the process were also analysed (Figure 11b). It can be observed that the temperature drops are relatively small and occur on the workpiece surface. It is worth noting that the pattern of variations in the temperature on the surface of produced balls is very similar to that obtained in the experimental tests. One can observe that the temperature increases above its initial value (up to 1050 °C, approximately) in the workpiece regions undergoing the highest plastic deformation, which is caused by the change of plastic deformation work and friction work into heat. However, in other regions of the ball, one can observe a systematic temperature drop, which is caused by the contact of the workpiece with the tools and the resulting transfer of heat to the environment.

Based on the distribution of the damage function according to the Cockcroft-Latham criterion (Figure 11c), crack occurrence during the rolling process was assessed as:

(3) $$C=\int_{0}^{\varepsilon }\frac{\sigma_{1}}{\sigma_{i}}d\varepsilon ,$$

where σ_1_ is the maximum principal stress, σ_i_ is the effective stress, *ε* is the strain, and *C* is the Cockcroft-Latham damage criterion.

It can be observed that the highest values of the damage function are concentrated in the region of the ball separation (in the region of connecting necks). However, in other zones (mainly in the centre of the ball), the values of the damage function are low. This behaviour pattern of the ductile damage criterion rules out the possibility of material coherence loss in the central regions of the balls. On the other hand, the very large concentration of the Cockcroft-Latham integral in the connecting neck region means that produced balls will be completely separated.

**Figure 12:**
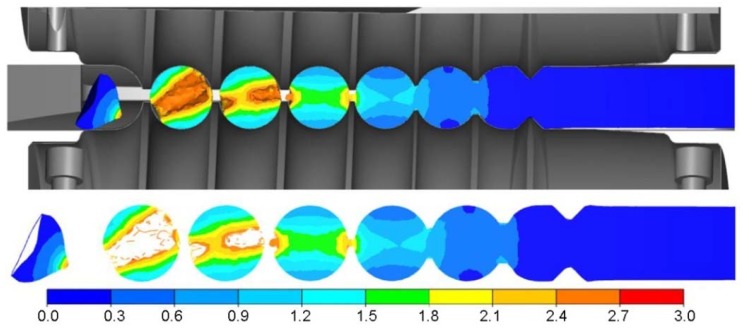
Distribution of the damage criterion according to Cockroft-Latham in the axial section of the semi product balls with a diameter bigger than nominal ∅41 mm.

Overfill caused by too great a diameter of the billet may lead to the material cracking, which is caused by the Mannesmann effect [20,21,22]. The phenomenon of cracking is complex and is assumed to be caused by the low cycle fatigue of the metal. During the processes of cross- and skew rolling, the material in the central zone of the forging is compressed in a direction normal to the tool contact surface, whereas it is tensed in a direction perpendicular to the normal. The strain state changes after the billet performs ¼ of a full rotation. The tensed zones are now being compressed, whereas the compressed ones are subject to tensing. Due to such a strain scheme fatigue cracks occur. This phenomenon is intensified as the differences in limit values of the central zone of the rolled tools increase. This is facilitated by the overfill of the impression as well as lengthening of the forming process, which causes an increase in the strain change cycles in the central zone. As far as rolling of 40 mm diameter balls from semi-finished product is concerned, the occurrence of Cockroft-Latham integral is inconsiderable and is limited to the separation zone (Figure 11c). Increasing the billet diameter (to ∅41 mm) causes a rapid increase of the extreme value of the damage criterion, as well as the expansion of their occurrence to the centre of the rolled parts (Figure 12). It is to be noted that the central zone of the balls is spanned by high values of the criterion. In the calculations it was assumed that the limit value of the criterion at which the material fails is equal to *C* = 3 [23]. At these values, the failure of the material occurs in the centre of the balls. Unfortunately, so far, no exact data concerning the method of determining the limit value of the damage criterion in the complex state of strain (among others for the processes of rotary forming) can be found. The information presented in the specialist literature mostly concerns simple cases of deformation (stretching and compression) [24,25,26,27]. Nevertheless, the criterion chosen can be used for reference purposes, allowing the estimation of the risk of the material cracking during the rolling process.

**Figure 13:**
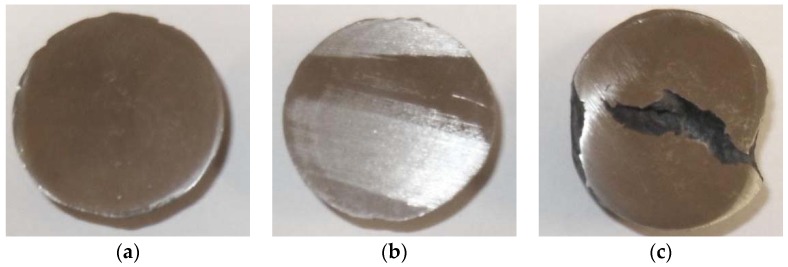
Axial sections of the balls in helical rolling manufactured from the semi-finished product of various diameter: (**a**) nominal ∅40 mm; (**b**) smaller than nominal ∅39 mm; (**c**) bigger than nominal ∅41 mm.

Interestingly enough, the relation noticed during the FEM simulation was confirmed by the results of the experimental tests (Figure 13). No cracking was detected in the cross-section of the balls (along the rolling axis) manufactured from the semi-finished product of the nominal diameter (∅40 mm) or of the smaller diameter (∅39 mm). However, in balls of a diameter bigger than the nominal (∅41 mm), a very extensive crack of the ball occurs in the central zone. On the basis of this information, it can be stated that the limit value of the Cockroft-Latham integral used in the calculations is correct and the results obtained approximate the real ones.

**Figure 14:**
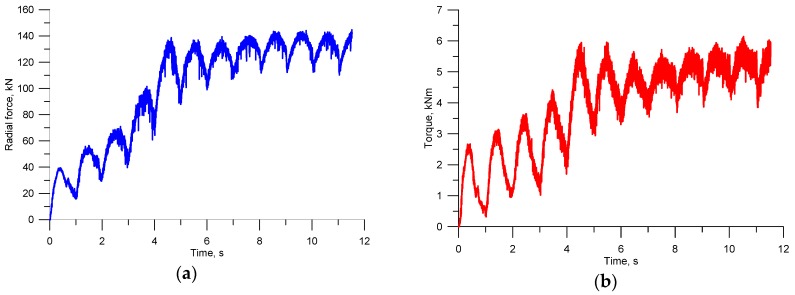
FEM-simulated variations in the force parameters in helical rolling of balls: (**a**) radial force; (**b**) torque.

The numerical modelling also involved an analysis of the variations in the forces and torques, as shown in Figure 14 (for the optimal parameters, diameter of billet ∅40 mm). A characteristic of the obtained force parameters is their periodic nature (as in the experimental tests), resulting from the periodic cutting of the wedge tools into the billet, every half revolution of the tools. It is visible that the initial stages of the process (the first two revolutions of the tools) are characterised by gradually increasing forces and torques, which results from the filling of successive roll passes with the material. Once the material has filled all helical roll passes of the tools, the maximum and minimum force parameters become stable. Comparing the FEM results and the experimental findings, one can notice their relatively high quantitative agreement (predominantly in relation to maximum values).

## 6. Summary and Conclusions

The obtained results indicate a significant impact of the billet parameters (temperature and diameter) used in helical rolling on the rolling process itself and the quality of produced balls. The diameter of billet material has the greatest impact on the quality of produced balls. Therefore, the correct tool design should enable the forming of balls with the required quality from steel rods manufactured in normal accuracy class. However, the rolling process strongly depends on the initial temperature of the billet. Billet preheating temperature should be selected such that balls can be subjected to thermal treatment immediately after rolling.

The results lead to the following conclusions:

- the highest geometric accuracy of balls is achieved when the balls are formed from the 40 mm-diameter billet (i.e., the billet diameter is 3.5% smaller than that of the ball);
- the use of the billet with a diameter 6% smaller than that of the ball leads to underfill; however, the achieved geometric accuracy of the rolled part is sufficient is the ball is to be used in grinding media;
- the use of billet materials with the same or bigger diameter as that of the balls causes overfill and serious surface defects of the balls;
- the billet should be preheated to the lowest possible temperature to enable the quenching of produced balls immediately after the rolling process;
- when the billet preheating temperature is too high, it is difficult to separate produced parts and the connecting necks between produced balls cannot be completely removed;
- overfill resulting from too great a billet diameter and the application of a low preheating temperature of the tool lead to a sudden increase in the force parameters, which results in a lower service life of the tools;
- the high agreement between the experimental findings and the FEM results proves that numerical modelling can be used for analysis of complex metal forming processes.

## Figures and Tables

**Table 1:** Basic specifications of the rolling mill.

Parameter	Value	Unit
Roll position in the mill stand	horizontal	-
Nominal diameter of the rolls	320	mm
Work length of the roll face	400	mm
Minimum roll space	300	mm
Maximum roll space	350	mm
Shaft turn angle	+/−12	deg
Minimum rotational speed of the rolls	15	rpm
Maximum rotational speed of the rolls	30	rpm
Nominal torque per roll (15 rev/min)	20	kNm
Nominal torque per roll (30 rev/min)	10	kNm
Overall dimensions of the machine	3.2 × 1.8 × 2.1	m
Machine weight	17,500	kg
Engine power	60/80	kW

**Table 2:** Dimensional accuracy of balls produced by helical rolling at different parameters.

Process/Billet Parameters	Ball Shape	Dimension *D_a_*, mm	Dimension *D_b_*, mm
*T* = 1000 °C *d_o_* = 39 mm	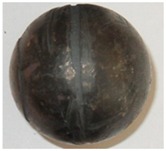	41.4	40.9
*T* = 1000 °C *d_o_* = 40 mm	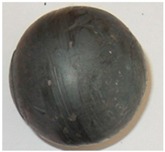	41.5	41.3
*T* = 1150 °C *d_o_* = 40 mm	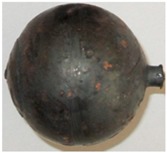	41.3	41.6
*T* = 1000 °C *d_o_* = 41 mm	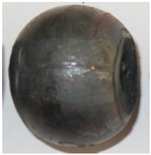	37.1	43.5